# Design and Synthesis of Arylthiophene-2-Carbaldehydes via Suzuki-Miyaura Reactions and Their Biological Evaluation

**DOI:** 10.3390/molecules181214711

**Published:** 2013-11-27

**Authors:** Shaukat Ali, Nasir Rasool, Aman Ullah, Faiz-ul-Hassan Nasim, Asma Yaqoob, Muhammad Zubair, Umer Rashid, Muhammad Riaz

**Affiliations:** 1 Department of Chemistry, Government College University, Faisalabad 38000, Pakistan;E-Mails: shaukat_ali_48@yahoo.com (S.A.); zubairmkn@yahoo.com (M.Z.); riaz_453@yahoo.com (M.R.); 2 Department of Agricultural, Food and Nutritional Science, University of Alberta, Edmonton, AB T6G2P5, Canada; 3 Department of Chemistry, Islamia University of Bahawalpur, Bahawalpur 63000, Pakistan; E-Mails: faiz.nasim@hotmail.com (F.N.); asmayaqoobctn@gmail.com (A.Y.); 4 Institute of Advanced Technology, Universiti Putra Malaysia, 43400 UPM Serdang, Selangor, Malaysia; E-Mail: umer.rashid@yahoo.com

**Keywords:** thiophene, Pd (0) catalyzed Suzuki coupling, antibacterial, heamolytic, antiurease, antioxidant, activities

## Abstract

A series of various novel 4-arylthiophene-2-carbaldehyde compounds were synthesized in moderate to excellent yields via Suzuki-Miyaura cross-coupling with different arylboronic pinacol esters/acids. The synthesized products were screened for their antibacterial, haemolytic, antiurease, and nitric oxide (NO) scavenging capabilities and interestingly, almost all products turned out to have good activities. 3-(5-Formyl-thiophene-3-yl)-5-(trifloromethyl)benzonitrile (**2d**) revealed excellent antibacterial activity, showing an IC_50_ value of 29.7 µg/mL against *Pseudomonas aeruginosa,* compared to the standard drug streptomycin with an IC_50_ value 35.2 µg/mL and was also found to be the best NO scavenger, with an IC_50_ value of 45.6 µg/mL. Moreover, 4-(3-chloro-4-fluoro-phenyl)thiophene-2-carbaldehyde (**2i**) exhibited a superior haemolytic action and an outstanding urease inhibition, showing an IC_50_ value of 27.1 µg/mL.

## 1. Introduction

In the recent years, Suzuki-Miyaura reactions have become one of the most powerful tools to build carbon-carbon bonds between weakly basic heterocyclic compounds and various arylboronic acids/esters [1,2]. These reactions have also been widely exploited in the synthesis of natural products [3,4], and the design of the pharmaceuticals [5]. C-O, C-N, and C-S bonds can also be constructed using these reactions due to their excellent functional group tolerance. Consequently, Suzuki cross-coupling reactions have acquired great importance in the synthesis of substituted aromatic compounds because these reactions are among the major and essential routes for any synthetic organic chemist. They are a well-established family of chemical reactions very useful for the synthesis of aryl-substituted thiophenes. Thiophene is used in advanced materials like dye-sensitized organic solar cells and light emitting devices [6], in luminescence, electron transport, redox activity, nonlinear optical chromism, electrochemical behavior, liquid crystalline characteristics and for its spectroscopic properties [7,8,9]. Thiophene-based molecules have shown numerous biological activities such as antitumor [10], analgesic [11], anti-inflammatory [12] and antibacterial activities [13], and as building blocks for agrochemicals [10]. Palladium-catalyzed functionalizations of thiophene derivatives have great importance due to the functional group compatibility, relative strength, low toxicity, and greater versatility of organopalladium complexes. Iqbal *et al.* [14], Al-Adiwish *et al.* [15], and Thomas *et al*. [16] reported that the arylthiophene derivatives bearing electron withdrawing substituents showed excellent antibacterial activity against Gram-negative bacteria. Furthermore, Sigmundova *et al.* [17] reported that arylthiophene derivatives with electron donating substituents exhibited potent antibacterial activity against Gram-positive bacteria. Relatively less attention has been paid to the synthesis of 4-arylthiophene-2-cabaldehydes and investigation of their biological activities, therefore, we focused our attention on the synthesis of various of these compounds. Moreover, the biological activities of these novel compounds were also studied to discover their potential to be used as new pharmaceutical agents. To the best of our knowledge, the antibacterial, antiurease, heamolytic, and antioxidant activities of these compounds have not been reported in the literature so far.

## 2. Results and Discussion

### 2.1. Chemistry

**Scheme 1:**
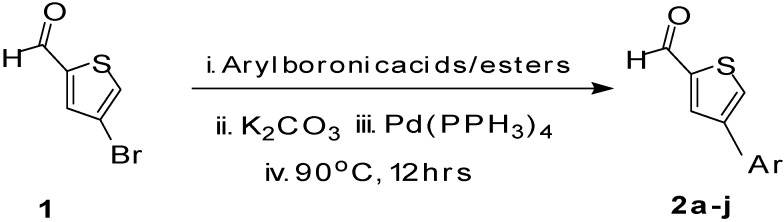
Synthesis of 4-arylthiophene-2-carbaldehydes **2a**–**j**.

Dang and coworkers [18] reported the Suzuki cross-coupling reaction of tetrabromothiophene with arylboronic acids to prepare the corresponding tetraarylthiophenes. Handy *et al.* [19] have reported double Suzuki couplings of 4,5-dibromothiophene-2-carbaldehyde with different arylboronic acids. Herein, we describe the application of the Suzuki cross-coupling reactions of 4-bromothiophene-2-carbaldehyde (**1**) with equimolar amounts of various arylboronic acids/esters in the presence of K_3_PO_4 _and Pd(PPh_3_)_4_ to form the corresponding 4-arylthiophene-2-carbaldehydes (Scheme 1).

The factors affecting the optimization of reactions were also studied. Thus, the effect of solvent on the coupling reactions between **1** and various arylboronic acids/esters under thermal conditions at 85–90 °C was evaluated (Table 1). 4-Phenylthiophene-2-carbaldehyde (**2a**) was obtained in good yield when **1** was coupled with phenylboronic ester in 4:1 toluene/water in the presence of 5 mol% of the Pd(0) catalyst using K_3_PO_4_ as base. Its ^1^H-NMR spectrum showed a multiplet in the δ 7.3–7.5 ppm region due to the aryl protons and two singlets at δ 7.8 ppm and δ 8.1 ppm due to the protons at the 3 and 5 positions in the thiophene ring, respectively. The aldehyde proton exhibited a single peak at δ 9.95 ppm. The mass spectrum of the product **2a** exhibited a molecular ion peak at *m/z* 188. Moreover, the smooth coupling of compound **1** with 3,5-trifloromethylphenylboronic ester in 4:1 dioxane/water under heating conditions gave 4-(3,5-bis(trifloromethyl)phenyl)thiophne-2-carbaldehyde (**2b**) in excellent yield. Its ^1^H-NMR spectrum revealed two singlets at δ 7.24 ppm and δ 7.84 ppm assigned to the protons at positions 5 and 3 of the thiophene ring, respectively. In addition, three singlets at δ 7.99, 8.06 and δ 9.99 ppm appeared due to the 2H at positions 2 and 6 of the aryl group, 1H at position 4 and the aldehyde proton, respectively. The mass spectrum of product **2b** exhibited a molecular ion peak at *m/z* 323. 5-Chloro-2,2-thiophene-5-carbaldehyde (**2c**) was obtained in 1,4-dioxane in 66% yield. Similarly, the cross coupling of **1** with formyltrifloromethylphenylboronic ester gave 3-(5-formylthiophene-3-yl)-5-(trifloromethyl)benzonitrile (**2d**) in moderate yield after refluxing in DMF. Reaction of **1** with *p*-tolylphenylboronic acid in the presence of potassium hydroxide at 90 °C yielded *p-*tolylphenyl-thiophene-2-carbaldehyde (**2e**). The ^1^H-NMR spectrum of this product gave a 3H singlet for the methyl group in the δ 2.37 ppm region and a multiplet for the phenyl ring protons in the δ 7.2–7.4 ppm region. The thiophene protons present at positions 3 and 5 also showed singlets at δ 7.9 ppm and δ 7.7 ppm, respectively. The singlet assigned to the aldehyde proton appeared at δ 9.94 ppm. The most efficient coupling of **1** with 3,5-dimethylphenylboronic acid produced 4-(3,5-dimethylphenyl)thiophene-2-carbaldehyde (**2g**) in excellent yield after heating for 12 h. Its ^1^H-NMR spectrum revealed a singlet at δ 2.35 ppm corresponding to the 6H of the two methyls present at positions 3 and 5 in the phenyl ring due to their similar environments. In addition, a singlet peak at δ 9.94 ppm was assigned to the aldehyde proton. The thiophene protons at positions 3 and 5 showed singlets at δ 8.0 and δ 7.79 ppm, respectively. The protons present at positions 2 and 6 of the aryl group gave a singlet at δ 7.2 ppm. The proton at the 4 position of the aryl group gave a singlet at δ 6.9 ppm. The mass spectrum of product **2g** exhibited a molecular ion peak at *m/z* 217. 5-Methyl-2,3-bisthiophene-5-carbalehyde (**2j**) exhibited a molecular ion peak at *m/z* 189.67. Its ^1^H-NMR spectrum revealed two doublets at δ 4.10 and δ 7.01 ppm due to the protons at the 3 and 5 positions in the thiophene ring, respectively. Moreover, two singlets appeared at δ 7.65 and δ 7.87 ppm due to the protons at positions 2 and 4 in the thiophene, respectively. The 3H of the methyl group also showed a singlet at δ 2.37 ppm. The aldehyde proton exhibited a singlet at δ 9.92 ppm. After the NMR and mass spectral study, these compounds were investigated for their biological activities.

**Table 1:** Synthesis of 4-arylthiophene-2-carbaldehydes.

Entry	ArylBoronic Acids/Esters	Products	Solvent/H_2_O (4:1)	Yields%
1	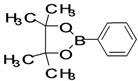	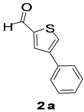	Toluene	67
2	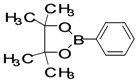	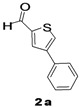	1,4-Dioxane	64
3	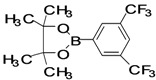	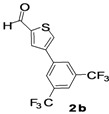	1,4-Dioxane	70
4	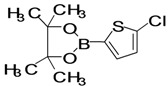	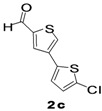	1,4-Dioxane	66
5	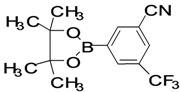	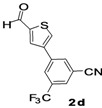	1,4-Dioxane	62
6	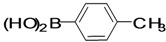	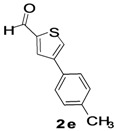	Toluene	64
7	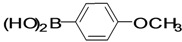	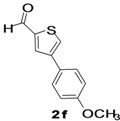	1,4-Dioxane	57
8	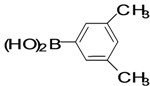	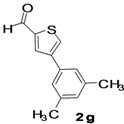	1,4-Dioxane	71
9	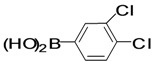	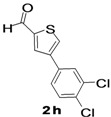	1,4-Dioxane	37
10	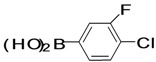	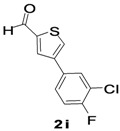	1,4-Dioxane	40
11	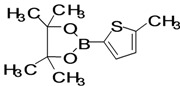	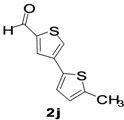	1,4-Dioxane	69

### 2.2. Biological Evaluation

#### 2.2.1. Antibacterial Activity

**Figure 1:**
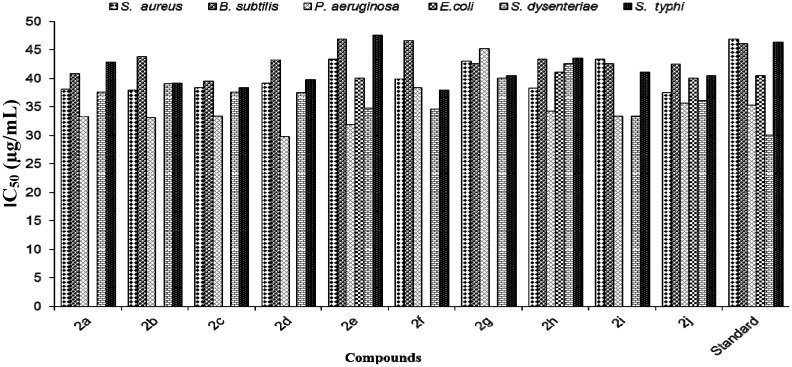
IC_50_ values of antibacterial activity.

Antibacterial activity of newly synthesized compounds **2a**–**j** was assayed against two selected strains of Gram-positive bacteria (*Staphylococcus aureus*, *Bacillus subtilis*) and four strains of Gram-negative bacteria (*Pseudomonas aeruginosa*, *Escherichia coli*, *Shigella dysenteriae*, *Salmonella typhi*) using the methods reported in the literature [20,21]. The plates were incubated for 24 h at 37 °C for bacteria. The activity against the organisms were tested with solutions of 25 and 50 µg/mL concentration and the resulting inhibition zone (IZ) was measured in mm as criterion for assessing the antibacterial activity. The test results at the two different concentrations are presented in Table 2 and Table 3 and further compared in Figure 1.

At the concentration of 25 µg/mL and 50 µg/mL, compound **2c** with an IC_50_ value of 39.4 µg/mL showed excellent activity against *Bacillus subtilis*. Compounds **2e**–**f**, however**,** expressed moderate activity against *Bacillus subtilis* showing IC_50_ values of 46.6 µg/mL, while, the compounds **2a**–**b**, **2d**, **2g**–**j** revealed mild to moderate activity when compared to the standard streptomycin. Compound **2d** showed outstanding inhibition against *Pseudomonas aeruginosa*, with an IC_50_ value 29.7 µg/mL, whereas compounds **2a**–**c**, **2e**–**f**, **2h**–**j** exhibited mild to average activity against *Pseudomonas aeruginosa.*

**Table 2:** Antibacterial activity of synthetic compounds at 25 µg/mL *.

Compound	Gram Positive Bacteria		Gram Negative Bacteria			
	*S. aureus*	*B. subtilis*	*P. aeruginosa*	*E.coli*	*S. dysenteriae*	*S. typhi*
**2b**	36.6 ± 0.4	23 ± 0.09	42 ± 0.02	18 ± 0.13	33 ± 0.05	33 ± 0.20
**2c**	34 ± 0.01	36.2 ± 0.05	45 ± 0.019	14 ± 0.14	35 ± 0.01	34 ± 0.05
**2d**	33 ± 0.01	28.3 ± 0.75	46 ± 0.02	32.5 ± 0.12	35 ± 0.15	30 ± 0.15
**2e**	28 ± 0.2	22.4 ± 0.13	44 ± 0.02	32 ± 0.05	41 ± 0.05	22.2 ± 0.14
**2f**	34 ± 0.05	24.4 ± 0.03	34 ± 0.04	9 ± 0.03	42 ± 0.7	36 ± 0.01
**2g**	27 ± 0.08	36 ± 0.08	25 ± 0.06	22.5 ± 0.11	32 ± 0.01	32.2 ± 0.11
**2h**	39.4 ± 0.05	28 ± 0.03	40 ± 0.041	32 ± 0.03	29 ± 0.19	30 ± 0.13
**2i**	27.8 ± 0.12	36.5 ± 0.04	42 ± 0.04	16 ± 0.043	43 ± 0.105	33 ± 0.25
**2j**	35.2 ± 0.01	27.10 ± 0.01	36 ± 0.16	26 ± 0.03	35 ± 0.2	29 ± 0.1
**Streptomycin**	15 ± 0.034	34 ± 0.015	41.5 ± 0.002	29.3 ± 0.002	48 ± 0.05	33 ± 0.001

* Inhibition zone (in mm); data are expressed in the form of mean ± SD.

**Table 3:** Antibacterial activity of synthetic compounds at 50 µg/mL *.

Compound	Gram Positive Bacteria		Gram Negative Bacteria			
	*S. aureus*	*B. subtilis*	*P. aeruginosa*	*E.coli*	*S. dysenteriae*	*S. typhi*
**2a**	63 ± 0.18	61 ± 0.14	67.6 ± 0.001	47 ± 0.15	63 ± 0.18	61 ± 0.13
**2b**	62.4 ± 0.1	59 ± 0.1	67 ± 0.01	38 0.18	62.4 ± 0.1	63 ± 0.25
**2c**	64 ± 0.11	60 ± 0.01	60 ± 0.009	24 ± 0.17	64 ± 0.11	64 ± 0.5
**2d**	63 ± 0.31	58 ± 0.35	67 ± 0.01	40.5 ± 0.19	63 ± 0.31	64 ± 0.05
**2e**	58 ± 0.01	54 ± 0.17	60 ± 0.03	62 ± 0.01	58 ± 0.01	53 ± 0.12
**2f**	61.4 ± 0.56	54 ± 0.23	64 ± 0.04	8 ± 0.01	61.4 ± 0.56	63 ± 0.11
**2g**	59 ± 0.44	56 ± 0.24	56 ± 0.04	44.5 ± 0.19	59 ± 0.44	61 ± 0.16
**2h**	59.4 ± 0.45	58 ± 0.22	67 ± 0.021	60 ± 0.24	59.4 ± 0.45	57 ± 0.01
**2i**	58 ± 0.16	56 ± 0.5	66 ± 0.01	26 ± 0.023	58 ± 0.16	59.5 ± 0.25
**2j**	65 ± 0.09	60 ± 0.05	69 ± 0.16	66 ± 0.43	65 ± 0.09	63 ± 0.1
**Streptomycin**	55 ± 0.03	53 ± 0.001	63 ± 0.002	63 ± 0.002	55 ± 0.034	53 ± 0.001

* Inhibition zone (in mm); data are expressed in the form of mean ± SD.

From the screening data, it was observed that electron withdrawing and donating groups on the aryl ring had a substantial influence on the antibacterial activities, confirming earlier reports that on the antibacterial activity of various aryl thiophene derivatives bearing electron donating and electron withdrawing groups [14,16,22]. It was previously claimed that the antibacterial activity of compounds containing electron withdrawing groups was more potent against Gram-negative bacteria. In agreement with that statement, in our case compound **2d** with CF_3 _and CN moieties and **2i** with 3,4-dichloro substitution on the phenyl ring showed potents result against Gram-negative bacteria. In [17], the authors concluded that compounds with electron donating groups were more potent against Gram-positive bacteria and showed non-significant activity against Gram-negative bacteria. In agreement with the literature reports, compound **2g** with a 3,5-dimethyl substitution on the phenyl ring gave very poor activity, with an IC_50_ value of 45.16 µg/mL against Gram-negative bacteria as compared to standard. Furthermore, **2j** showed excellent antibacterial activity against *Staphylococcus aureus*, with an IC_50_ value of 37.4 µg/mL, whereas, the compounds **2a**–**i** exhibited mild to moderate activities against *Staphylococcus aureus* when compared to the standard. In addition, **2e** and **2j** were found to be active against *Escherichia coli*, with IC_50_ values of 40 µg/mL. Nevertheless, **2a**–**d**, **2f**–**i** were found to be inactive with zero IC_50_ value against *Escherichia coli.* Compound **2j** with an IC_50_ value of 37.4 µg/mL showed excellent activity against *Salmonella typhi.* Compound **2f** with a 4-methoxyphenyl ring was found to be the most active compound against the Gram-negative bacterium *Salmonella typhi*, with an IC_50_ value 37.9 µg/mL, but the main contribution to the activity probably came from the oxygen moiety of the methoxy group on the phenyl ring.

#### 2.2.2. Antiurease Activity

**Figure 2:**
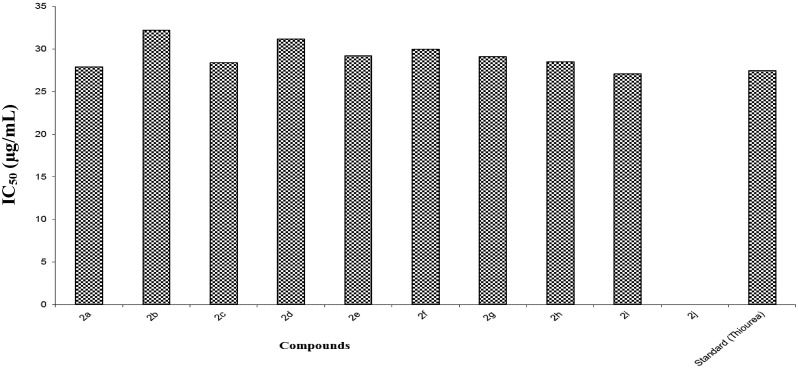
IC_50_ values of anti-urease activity.

Antiurease activity testing was carried out in accordance with the literature protocol [23] by using thiourease, with an IC_50_ value of 27.5 µg/mL, as the standard inhibitor. The synthesized compounds **2a**–**j** were examined for their urease inhibition activity at 25 µg/mL and 50 µg/mL concentration (Table 4, Figure 2). It was considered that all these compounds could have the ability to bind with the enzyme’s active site, whereby the enzyme stops catalyzing hydrolysis and the enzyme activity is inhibited. All synthesized compounds showed moderate to excellent urease inhibition. The compounds **2a** and **2i** proved to be the most potent urease inhibitor, with IC_50_ values of 27.9 µg/mL and 27.1 µg/mL, respectively. These values were comparable to the 27.5 µg/mL value of the thiourea standard. Molecules **2b**–**h** exhibited comparatively moderate antiurease activities. It was observed that compound **2j** was inactive against urease enzyme, with an IC_50_ value of 0 µg/mL. Noteworthy, compound **2e** gave good urease inhibition, with an IC_50_ value of 29.2 µg/mL at 50 µg/mL. Notably, we observed that various functional groups present on aryl group were responsible for variable urease inhibitions. The compound **2i** with 3-chloro, 4-floro groups lead to enhanced urease inhibition, while the compounds **2b**, **2f** with electron donating groups exhibited less urease inhibition, although, compound **2d** with electron withdrawing CN functional group showed surprisingly poor urease inhibiting action.

**Table 4:** Antiurease activity of synthesized compounds.

Entry	%age Activity at 25 μg	%age Activity at 50 μg	IC_50_ (µg/mL)
**2a**	45 ± 0.0162	86.9 ± 0.012	27.9
**2b**	35 ± 0.014	87.0 ± 0.084	32.2
**2c**	44 ± 0.0042	87.8 ± 0.006	28.4
**2d**	37 ± 0.0042	88.7 ± 0.005	31.2
**2e**	44.5 ± 0.057	76.5 ± 0.05	29.2
**2f**	42.3 ± 0.0007	80.2 ± 0.0017	30.0
**2g**	42.5 ± 0.0070	87.2 ± 0.5	29.1
**2h**	44 ± 0.084	86.4 ± 0.44	28.5
**2i**	46.2 ± 0.0042	90.6 ± 0.05	27.1
**2j**	2 ± 0.219	9.7 ± 0.2	Zero
**Thiourea**	47 ± 0.007	77 ± 0.015	27.5

#### 2.2.3. Nitric Oxide (NO) Scavenging Assay

Nitric oxide (NO) is a free radical causing inflammatory disorders and cancer in the human body when sodium nitroprusside decomposes to produce nitric oxide [24,25]. The antioxidant activity of various thiophene derivatives has been reported in the literature and it has also been observed that compounds with high antioxidant activity served as antitumor agents [26]. The newly synthesized compounds reacted directly with nitric oxide and inhibited nitrite formation in an excellent way. The compounds **2a**–**j** were evaluated for their nitric oxide scavenging activity according to the literature protocol [27], at concentrations of 25, 50 and 100 µg/mL, respectively, and ascorbic acid (vitamin C) was used as standard. Ascorbic acid showed a 40% ± 0.038% NO scavenging activity at 25 µg/mL, 61 ± 0.004 at 50 µg/mL and 77 ± 0.001 at 100 µg/mL concentrations, respectively (Table 5).

**Table 5:** NO scavenging activity of synthesized compounds.

Compound	% Activity at 25 µg	% Activity at 50 µg	% Activity at 100 µg	IC_50 _µg/mL
**2a**	12 ± 0.037	33 ± 0.0035	48 ± 0.049	92.5
**2b**	10 ± 0.02	45 ± 0.001	60 ± 0.021	66.6
**2c**	18 ± 0.007	40 ± 0.038	55 ± 0.040	83.3
**2d**	12.0 ± 0.025	58 ± 0.010	73 ± 0.0028	45.6
**2e**	−15 ± 0.04	−12 ± 0.037	3 ± 0.0014	NA
**2f**	5 ± 0.005	10 ± 0.028	25 ± 0.005	NA
**2g**	13 ± 0.002	28 ± 0.007	43 ± 0.002	NA
**2h**	13 ± 0.002	20 ± 0.025	35 ± 0.077	NA
**2i**	12 ± 0.05	57 ± 0.0021	72 ± 0.009	46.1
**2j**	15 ± 0.021	50 ± 0.024	65 ± 0.013	49.3
**Ascorbic Acid**	40 ± 0.038	61 ± 0.004	77 ± 0.001	56.33

The compounds **2d** and **2i** when systematically analyzed were found to be the most active in NO scavenging, with percent activities of 58 ± 0.010 and 57 ± 0.0021 at 50 µg/mL and IC_50_ values of 45.6 µg/mL and 46.1 µg/mL, respectively. Compound **2j** expressed comparatively excellent activity showing a NO scavenging percentage 50 ± 0.024 at 50 µg/mL and 65 ± 0.013 at 100 µg/mL and an IC_50_ value of 49.3 µg/mL. It was noted that compound **2b** also exhibited a significant NO scavenging activity, with percentages of 45 ± 0.001 at 50 µg/mL and 60 ± 0.021 at 100 µg/mL and an IC_50_ value of 66.6 µg/mL. Moderate NO scavenging percentages of 33 ± 0.0035 and 40 ± 0.038 at 50 µg/mL were observed for **2a** and **2c**, with IC_50_ values 92.5 µg/mL and 83.3 µg/ML, respectively, whereas, the compounds **2e**–**h** were found to be inactive for NO scavenging. Based on above results, we conclude that different functional groups present on 4-arylthiophene-2-carbaldehyde derivatives **2a**–**j** had substantial effects on the percent NO scavenging activities. It was surprisingly observed that compound **2i** with 3-chloro, 4-fluoro moiety showed a higher percentage of NO scavenging.

#### 2.2.4. Haemolytic Activity

The cytotoxicity to blood lymphocytes, thymocytes and spleen cells of various thiophene derivatives has already been reported [28]. The newly synthesized compounds **2a**–**j** were also studied for haemolytic activity by the method used by Sharma and Powell [29,30]. The compounds **2f** and **2i** exhibited the highest haemolytic action, but less than the positive control (Triton-X 100), which showed the maximum percentage of lysis (99.824 ± 0.536). Compounds **2d** and **2e** displayed the lowest haemolytic property, but higher than the negative control PBS which showed zero percentage lysis (Table 6). Compounds **2a**–**c**, **2g**–**h**, **2j** demonstrated moderate haemolytic activity. The compounds which showed the highest haemolytic activity might be considered as potential antitumor agents.

**Table 6:** Haemolytic activity of synthetic compounds **2a**–**j**.

Entry	Average ± SD	Entry	Average ± SD
**2a**	3.947 ± 0.078	2f	6.083 ± 0.143
**2b**	5.003 ± 0.078	2g	4.525 ± 0.075
**2c**	3.934 ± 0.095	2h	3.487 ± 0.054
**2d**	3.306 ± 0.058	2i	9.779 ± 0.095
**2e**	3.356 ± 0.075	2j	4.135 ± 0.095
**Standard**		99.824 ± 0.536	

## 3. Experimental

### 3.1. General

Melting points were determined using a Buchi Melting Point B-540 apparatus. All reagents were purchased from Alfa Aesar Chemical Co. (St. Parkridge Ward Hill, MA, USA) and Sigma Aldrich Chemical Co. (St. Louis, MO, USA). ^1^H-NMR and ^13^C-NMR spectra were measured in CDCl_3_ and CD_3_OD on a Bruker Aspect AM-400 instrument at 400/100 MHz The chemical shift was given in δ in ppm and coupling constant in Hz*.* EI-MS spectra were recorded on a JMS-HX-110 spectrometer with a data system. For column chromatography technique, silica gel (70–230 mesh) and silica gel (230–400 mesh) were used. The reactions were monitored on TLC, using Merck silica gel 60 PF_254 _cards. The compounds were visualized by UV lamp (254–365 nm).

### 3.2. General Procedure of Synthesis of 4-Arylthiophene-2-Carbaldehyde

To 4-bromothiophene-2-carbaldehdyde (**1**, 0.704 mmol) 5% mol Pd(PPh_3_)_4_ was added in 1,4-dioxane under argon atmosphere. The reaction mixture was stirred at room temperature for 30 min. Arylboronic esters/acids (0.774 mmol), and potassium phosphate (1.409 mmol) were added along with water (1.5 mL) under an argon atmosphere. The solution was stirred at 90 °C for 12 h and then cooled to room temperature. The organic layer was extracted using ethyl acetate, decanted and dried over magnesium sulphate. Then the solvent was removed under reduced pressure. The crude residue obtained was purified by column chromatography using *n*-hexane and ethyl acetate in 1:1 ratio to obtain the desired products, which were characterized by spectroscopic techniques.

### 3.3. Characterization Data

*4-Phenylthiophene-2-carbaldehyde* (**2a**). Mp: 57–59 °C; ^1^H-NMR (CDCl_3 _+ CD_3_OD): δ = 9.95 (s, 1H), 8.01 (s, 1H-thiophene), 7.83 (s, 1H-thiophene), 7.5 (d, *J* = 1.5 Hz, 1H-aryl), 7.44–7.39 (m, 2H-aryl), 7.36–7.31 (m, 2H-aryl). ^13^C-NMR (CDCl_3 _+ CD_3_OD): δ = 126.30, 127.19, 128.09, 128.77, 129.06, 129.63, 130.86, 134.34, 134.73, 143.64, 144.37, 183.01; EIMS (*m/z* +ion mode): 188 [M‒H^+^]; [M‒CHO]^+^ = 160.01; [M‒C_7_H_6_]^+^ = 102.1; [M‒phenyl]^+^ = 115.1. Anal. Calcd. for C_11_H_8_OS (188.03): C, 70.18; H, 4.28; O, 8.50; S, 17.03. Found: C, 70.22; H, 4.26; O, 8.55; S, 17.12%.

*4-(3,5-Bis(trifloromethyl)phenyl)thiophene-2-carbaldehyde* (**2b**). Mp: 95–97 °C; ^1^H-NMR (CDCl_3_ + CD_3_OD): δ = 9.99 (s, 1H), 8.06 (s, 1H-aryl), 7.99 (s, 2H-aryl), 7.84 (s, 1H-thiophene), 7.24 (s, 1H-thiophene).^ 13^C-NMR (CDCl_3 _+ CD_3_OD): δ = 121.57, 126.32, 131.31, 132.37, 132.81, 133.76, 136.42, 140.44, 145.39, 182.56; EIMS (*m/z* +ion mode): 323 [M‒H^+^]; [M‒2C]^+ ^= 226.0; [M‒O]^+ ^= 207; [M‒F_2,_C_7_H_6_O]^+^ = 182.1; [M‒Ph‒3-CF_3_‒5-CF_3_]^+^ = 113. Anal. Calcd. for C_13_H_6_F_6_OS (324.00): C, 48.16; H, 1.87; F, 35.16; O, 4.93; S, 9.89. Found: C, 48.22; H, 1.93; F, 35.05; O, 4.81; S, 9.78%.

*5-Chloro-2,2-bisthiophene-5-carbaldehyde* (**2c**). Mp: 229–231 °C; ^1^H-NMR (CDCl_3 _+ CD_3_OD): δ = 9.84 (s, 1H), 8.00 (d, *J* = 1.1 Hz, 1H-thiophene), 7.83 (d, *J* = 1.2 Hz, 1H-thiophene), 7.51 (d, *J* = 6.9 Hz, 1H-thiophene), 6.7 (d, *J* = 3.9 Hz, 1H-thiophene). ^13^C-NMR (CDCl_3_ + CD_3_OD): δ = 123.4, 125.2, 126.3, 127.5, 136.7, 138.1, 140.2, 143.4, 182.4; EIMS (*m/z* +ion mode): 229 [M‒H^+^]; [M‒CHO]^+^ = 201.00; [M‒O]^+^ = 211.00; [M‒thiophene]^+^ = 147.17. Anal. Calcd. for C_9_H_5_OS_2_ (192.98): C, 55.92; H, 2.61; O, 8.28; S, 33.18. Found: C, 55.97; H, 2.65; O, 8.32; S, 33.13%.

*3-(5-Formylthiophene-3-yl)-5-(trifloromethyl)benzonitrile* (**2d**). Mp: 252–254 °C; ^1^H-NMR (CDCl_3_ + CD_3_OD): δ = 9.97 (s, 1H), 8.08 (s, 1H-aAryl), 8.03 (s, 1H-aryl), 7.61–7.53 (m, 2H-thiophene, 1H-aryl). ^13^C-NMR (CDCl_3_ + CD_3_OD): δ = 22.69, 116.97, 127.09, 129.65, 131.54, 132.79, 133.40, 134.69, 137.95, 143.65, 144.30, 182.98; EIMS (*m/z* +ion mode): 280.58 [M‒H^+^]; [M‒CN]^+^ = 256; [M‒F_2_]^+^ = 243.91; [M‒thiophene]^+^ = 198.70. Anal. Calcd. for C_13_H_6_F_3_NOS (281.05): C, 55.52; H, 2.15; F, 20.26; N, 4.98; O, 5.69; S, 11.40. Found: C, 55.57; H, 2.19; F, 20.21; O, 5.64; S, 11.48%.

*4-p-Tolylthiophene-2-carbaldehyde* (**2e**). Mp: 58–60 °C; ^1^H-NMR (CDCl_3 _+ CD_3_OD): δ = 9.94 (s, 1H), 7.99 (s, 1H-thiophene), 7.791 (s, 1H-thiophene), 7.4 (d, *J* = 7.5 Hz, 1H-aryl), 7.2 (d, *J* = 7.8 Hz, 1H aryl), 2.37 (s, 3H). ^13^C-NMR (CDCl_3_ + CD_3_OD): δ = 22.67, 126.18, 129.06, 129.74, 131.58, 134.69, 137.95, 143.65, 144.30, 182.98; EIMS (*m/z* +ion mode): 202.01 [M‒H^+^]; [M‒CH_3_]^+^ = 187.05; [M‒CHO]^+^ = 173.98; [M‒Ph]^+^ = 111.76; [M‒thiophenecarbaldehyde]^+^ = 77.58. Anal. Calcd. for C_12_H_10_OS (202.01): C, 17.25; H, 4.98; O, 7.91; S, 15.85. Found: C, 17.28; H, 4.92; O, 7.93; S, 15.82%.

*4-(4-Methoxyphenyl)thiophene-2-carbaldehyde* (**2f**). Mp: 68–70 °C; ^1^H-NMR (CDCl_3_ + CD_3_OD): δ = 9.84 (s, 1H), 8.16 (s, 1H-thiophene), 8.06 (s, 1H-thiophene), 7.68 (m, 2H-aryl), 7.05 (d, *J* = 8.4 Hz, 2H-aryl), 3.83 (s, 3H-OCH_3_).^ 13^C-NMR (CDCl_3_ + CD_3_OD): δ = 55.38, 114.48, 127.72, 128.63, 132.18, 134.58, 143.38, 144.28, 159.57, 183.01; EIMS (*m/z* +ion mode): 217.86 [M‒H^+^]; [M‒CHO]^+^ = 190.56; [M‒OCH_3_]^+^ = 188.03; [M‒Ph]^+^ = 141.76; [M‒thiophenecarbaldehyde]^+^ = 108.07. Anal. Calcd. for C_12_H_10_O_2_S (218.02): C, 66.03; H, 4.62; O, 14.66; S, 14.69. Found: C, 66.12; H, 4.66; O, 14.59; S, 14.72%.

*4-(3,5-Dimethylphenyl)thiophene-2-carbaldehyde* (**2g**). Mp: 71–73 °C; ^1^H-NMR (CDCl_3_ +CD_3_OD): δ = 9.94 (s, 1H), 8.00 (s, 1H-thiophene), 7.79 (s, 1H-thiophene), 7.18 (s, 2H-aryl), 6.98 (s, 1H-aryl), 2.35 (s, 6H, 2CH_3_). ^13^C-NMR (CDCl_3_ + CD_3_OD): δ = 21.35, 124.24, 129.47, 129.70, 134.28, 134.98, 138.66, 143.91, 144.23, 183.01; EIMS (*m/z* +ion mode): 217 [M‒H^+^]; [M‒CHO]^+^ = 189.00; [M‒2CH_3_]^+^ = 189.00. Anal. Calcd. for C_13_H_12_OS (216.06): C, 72.19; H, 5.59; O, 7.40; S, 14.82. Found: C, 72.24; H, 5.62; O, 7.38; S, 14.85%.

*4-(3,4-Dichlorophenyl)thiophene-2-carbaldehyde* (**2h**). Mp: 158–160 °C; ^1^H-NMR (CDCl_3_ + CD_3_OD):δ = 9.95 (s, 1H), 7.94 (s, 1H-thiophene), 7.79 (s, 1H-thiophene), 7.62 (s, 1H-Aryl), 7.59 (d, *J* = 2.0 Hz, 1H-Aryl), 7.42 (d, *J* = 2.4 Hz, 1H-Aryl). ^13^C-NMR (CDCl_3 _+ CD_3_OD): δ = 117.32, 121.87, 126.10, 129.91, 131.74, 134.15, 141.31, 144.81, 156.63, 159.12, 182.72; EIMS (*m/z* +ion mode): 256 [M‒H^+^]; [M‒CO]^+^ = 229.17.00; [M‒thiophene]^+^ = 85.00. Anal. Calcd. for C_11_H_6_Cl_2_OS (255.96): C, 51.38; H, 2.35; Cl, 27.58; O, 6.22; S, 12.47. Found: C, 51.40; H, 2.38; Cl, 27.63; O, 6.18; S, 12.51%.

*4-(3-Chloro-4-fluorophenyl)thiophene-2-carbaldehyde* (**2i**). Mp: 108–110 °C; ^1^H-NMR (CDCl_3 _+ CD_3_OD): δ = 9.95 (s, 1H), 7.83 (s, 1H-thiophene), 7.50 (s, 1H-thiophene), 7.48 (s, 1H-aryl), 7.41 (d, *J* = 2.0 Hz, 1H-Aryl), 7.39 (d, *J* = 2.0 Hz, 1H-aryl). ^13^C-NMR (CDCl_3_ + CD_3_OD): δ = 117.20, 126.07, 128.53, 129.90, 131.73, 134.13, 141.31, 144.81, 156.63, 159.12, 182.71; EIMS (*m/z* +ion mode): 240.25 [M‒H^+^]; [M‒CHO]^+^ = 213.08; [M‒F]^+^ = 195.08. Anal. Calcd. for C_11_H_6_ClFOS (239.98): C, 54.89; H, 2.51; Cl, 14.73; F, 7.89 O, 6.65; S, 13.32. Found: C, 54.93; H, 2.51; Cl, 14.69; F, 7.91; O, 6.59; S, 13.36S%.

*5-Methyl-2,3-bisthiophene-5-carbaldehyde* (**2j**). Mp: 218–220 °C; ^1^H-NMR (CDCl_3 _+ CD_3_OD): δ = 9.92 (s, 1H), 7.87 (s, 1H-thiophene), 7.65 (s, 1H-thiophene), 7.01 (d, *J* = 3.2 Hz, 1H-thiophene), 4.10 (d, *J* = 6.8 Hz, 1H-thiophene), 2.02 (s, 3H-methyl). ^13^C-NMR (CDCl_3 _+ CD_3_OD): δ = 15.34, 21.02, 22.67, 29.68, 60.38, 123.92, 126.05, 127.70, 133.91, 182.81; EIMS (*m/z* +ion mode): 189.67 [M‒H^+^]; [M‒CH_3_]^+^ = 175.87; [M‒CO]^+^ = 161.67; [M-thiophene]^+^ = 107.75. Anal. Calcd. for C_10_H_8_OS_2_ (208.02): C, 57.66; H, 3.37; O, 7.68; S, 30.79. Found: C, 57.61; H, 3.42; O, 7.64; S, 30.82%.

### 3.4. General Antibacterial Activity Assay Procedure

The newly synthesized compounds **2a**–**j** were screened for their antibacterial activity through microplate reader-based high throughput screening method [31] against four different strains of Gram-negative bacteria (*Pseudomonas aeruginosa*, *Escherichia coli*, *Shigella dysenteriae*, *Salmonella typhi*) and two strains of Gram-positive bacteria (*Staphylococcus aureus*, *Bacillus subtilis*). These strains were obtained from the Agha Khan University, Karachi, Pakistan. The standard antibiotic discs were purchased from the manufacturer (Oxoid Ltd, Basingstoke, UK). The synthesized compounds were dissolved in a solvent to get solutions of known concentration (5 µg/mL and 10 µg/mL). Using Whatman filter paper (No. 44), two discs of 5 mm diameter and 0.65 mm thickness were cut and sterilized in a hot air oven. In order to prepare sample discs of different concentrations, known volumes of compounds solutions were applied on the discs, air dried and used. The standard antibiotic discs were also prepared in the same way and used as positive control. Similarly, a disc prepared by applying only solvent was used as negative control. The glass apparatus used for the antibacterial activity was washed with plenty of water and sterilized in a hot air oven at 137 °C for 30 min. The sterilized nutrient agar medium was poured under aseptic conditions into sterile glass petri dishes/plates. For the maintenance of sub-cultures inoculation from stock cultures into nutrient broth medium poured either in test tubes or flasks was done and cultures were grown over night for 16 h at 37 °C on shaker. After inoculation petri plates were kept at room temperature for 30 min so that the poured medium is adsorbed. All the discs, including test sample, positive and negative controls were placed on inoculated nutrient agar medium solidified in petri dishes with the help of sterile forceps and the petri plates were incubated at 37 °C ± 2 °C for 24 h. Zone of inhibition was measured in mm using an ordinary ruler.

### 3.5. Antiurease Activity Assay Procedure

The reaction mixtures comprising sample solution (2 µL), phosphate buffer (17 µL) and sodium nitroprusside (20 µL) were incubated at 37 °C for 2 h in well plates. Then Griess reagent (20 µL, 0.3% sulphanilic acid in glacial acetic acid + 0.1% naphthylethylenediamine (NED): equal volumes of both solutions mixed just before use) was added to each well plate and the mixture kept at room temperature for 20 min to develop the color. The absorbance was measured at 528 nm by using an ELISA reader. The results were processed using Gen 5 software. All the reactions were performed in triplicate. Positive control (containing known antioxidant) and control (containing all samples) were run in parallel. Negative control was used to calibrate the instrument. The % age antioxidant activity was determined by using the following formula:

% antioxidant activity = [(Absorbance of control − absorbance of sample)/(Absorbance of control)] × 100

### 3.6. Haemolytic Activity Assay Procedure

The newly synthesized compounds **2a**–**j** were studied for haemolytic activity [32]. Three mL of freshly obtained human blood was placed in heparinized tubes to avoid coagulation, gently mixed and poured into a sterile 15 mL Falcon tube and centrifuged for 5 min at 1,000 *×g*. The supernatant was discarded and red blood cells were washed three times with chilled (4 °C) sterile isotonic phosphate buffer saline pH 7.4 (5 mL). Erythrocytes (10^8^ cells/mL) were maintained for each assay by dilution with phosphate buffer saline. Erythrocytes were counted on a heamacytometer. Twenty µL of each compound was mixed with red blood cells suspension (180 μL) separately. The samples were incubated at 37 °C for 35 min. The samples were placed on ice for 5 min and then agitated at 1,000 *×g* for 5 min. Supernatant (100 µL) were taken from each tube and diluted 10 times with chilled (4 °C) phosphate buffered saline. Triton X-100 (0.1% v/v) was taken as a positive control and PBS was negative control and then subjected to the same process. The absorbance was noted at 576 nm by using a Quant instrument (BioTek, Winooski, VT, USA). The percentage RBSs lyses for each sample was calculated.

## 4. Conclusions

Suzuki-Miyaura reactions were successfully exploited to synthesize a series of 4-arylthiophene-2-carbaldehyde **2a**–**j**. The reaction progress and purity of all synthesized compounds were monitored by TLC and products were characterized and their structure confirmed by NMR, elemental analysis, mass spectroscopy and melting points. It was observed from the results of antibacterial activity, heamolytic activity, urease inhibition and NO scavenger property that both electron withdrawing and electron donating groups on aryl ring substantially influenced the activities. The compounds possessing electron withdrawing groups on aryl ring exhibited potent antibacterial action against Gram-negative bacteria and those with electron donating group on aryl ring showed excellent antibacterial activity against Gram-positive bacteria. To evaluate the efficiency of the various 4-arylthiophene-2-cabaldehyde derivatives, we compared different functional groups present at various positions on aryl ring. Compound **2d** emerged as the most active against Gram-negative bacteria and was also found to be an outstanding NO scavenger, while the remaining compounds also exhibited mild to moderate activities when compared to the standards. The antiurease and the heamolytic screening results indicated that compound **2i** was an excellent antiurease and heamolytic agent, while the other compounds showed mild to significant activities as compared to the standards employed.
